# Development and Characterization of Film-Forming Solution Loaded with *Syzygium cumini* (L.) Skeels for Topical Application in Post-Surgical Therapies

**DOI:** 10.3390/pharmaceutics16101294

**Published:** 2024-10-04

**Authors:** Rosinéia Aparecida Vilela Cebrian, Mariana Dalmagro, Mariana Moraes Pinc, Guilherme Donadel, Larissa Aparecida Engel, Reinaldo Aparecido Bariccatti, Rafael Menck de Almeida, Kelen Menezes Flores Rossi de Aguiar, Emerson Luiz Botelho Lourenço, Jaqueline Hoscheid

**Affiliations:** 1 Laboratory of Preclinical Research of Natural Products, Paranaense University (UNIPAR), Umuarama 87502-210, Brazil; rosineia.cebrian@edu.unipar.br (R.A.V.C.); mariana.dal@edu.unipar.br (M.D.); mariana.pinc@edu.unipar.br (M.M.P.); g.donadel@edu.unipar.br (G.D.); larissa.engel@edu.unipar.br (L.A.E.); emerson@prof.unipar.br (E.L.B.L.); 2 Center for Engineering and Exact Sciences, State University of Western Paraná, Toledo 85903-220, Brazil; reinaldo.bariccatti@unioeste.br; 3 Synthetica Research and Technical Analysis Ltd., Capela do Alto 18195-000, Brazil; rafaelmenck@synthetica.com.br; 4 Group of Polymers and Nanostructures, Federal Technological University of Paraná, Toledo 85902-490, Brazil; kelenaguiar@utfpr.edu.br

**Keywords:** healing, jambolão, skin diseases, skin health, topical use

## Abstract

Background/Objectives: Considering the antioxidant and antimicrobial properties attributed to compounds in *Syzygium cumini* extract, this research aimed to advance postoperative therapeutic innovations. Specifically, the study assessed the physicochemical properties of a film-forming solution (FFS) incorporated with *S. cumini*, evaluating its therapeutic potential for postoperative applications. Methods: The *S. cumini* extract was meticulously characterized to determine its chemical composition, with particular emphasis on the concentration of phenolic compounds. Antioxidant and antimicrobial assays were conducted to assess the extract’s efficacy in these domains. Following this, an FFS containing *S. cumini* was formulated and evaluated comprehensively for skin adhesion, mechanical and barrier properties, and thermal behavior. Results: The antioxidant and antimicrobial activities of the *S. cumini* extract demonstrated promising results, indicating its potential utility as an adjunct in postoperative care. The developed FFS exhibited favorable physicochemical properties for topical application, including adequate skin adhesion and appropriate pH levels. Moreover, chemical and thermal analyses confirmed the formulation’s stability and the retention of the extract’s beneficial properties. Conclusions: Overall, the findings suggest that the *S. cumini*-loaded FFS holds significant potential as a valuable therapeutic tool for post-surgical management.

## 1. Introduction

The rising incidence of emerging infectious diseases, cancer, and multi-resistant pathogens has intensified the search for novel plant-derived molecules for pharmaceutical development [1]. The current literature demonstrates that certain medicinal plants and their phytotherapeutic formulations can promote tissue regeneration in both in vivo and in vitro models, including excisional and incisional experimental models [2,3,4,5].

While a single synthetic compound may exhibit stronger antioxidant and antimicrobial effects, plant extracts contain a complex mixture of compounds that act synergistically, enhancing the antimicrobial, antioxidant, anti-inflammatory, and immunomodulatory effects [6,7]. Thus, these plants also exhibit protective properties against wound infections and stimulate tissue regeneration by enhancing the immune system [8,9]. Furthermore, approximately one-third of available phytotherapeutics are employed in the treatment of diseases and the healing of skin lesions [10].

Another advantage is the chemical complexity of plant extracts, which includes secondary metabolites such as flavonoids, terpenoids, and alkaloids. These compounds can target multiple biological pathways, thereby reducing the risk of bacterial resistance. Furthermore, many of these substances possess antioxidant properties, which are critical in cosmetics to mitigate the damage caused by reactive oxygen species (ROS), which can hinder the healing process [6,11,12].

The stratum corneum of the epidermis facilitates the application of topical medications, including plant extracts, whose efficacy is closely related to the physicochemical properties of their constituents. Consequently, the incorporation of plant extracts into topical formulations could provide an alternative to or could complement synthetic drugs in postoperative treatments [13].

Transdermal drug delivery offers several advantages, including reduced side effects, sustained and non-invasive administration, and ease of application. Traditionally, drug delivery has been made through ointments, creams, and patches, which have inherent limitations. A promising alternative is the use of film-forming solution (FFS) [8,14].

FFS delivers the drug in a polymeric solution that forms a film at the application site. This thin film enhances drug concentration, approaching supersaturation at the site, thereby increasing the thermodynamic activity of the formulation, facilitating penetration through the dermal barrier and reducing irritation and side effects [15].

*Syzygium cumini* (L.) Skeels is an evergreen tree belonging to the Myrtaceae family [16]. Its pulp is rich in bioactive compounds, including flavonoids, triterpenes, sterols, and phenolic acids [17]. These compounds confer potent antioxidant, anti-inflammatory, and antitumor properties to *S. cumini*, lowering the risk of various diseases [18]. Additionally, *S. cumini* exhibits significant antimicrobial activity, demonstrating efficacy against a range of bacterial and fungal pathogens, thus supporting its use in the prevention and treatment of infections [19].

Given the well-documented bioactive properties of *S. cumini*, particularly in relation to its anti-inflammatory and antioxidant effects [20,21,22], and considering that excessive ROS production during the inflammatory phase can hinder wound healing [23], this study aims to develop an FFS loaded with *S. cumini* extract (FFS *S. cumini*) for topical application in postoperative care.

## 2. Materials and Methods

### 2.1. Plant Material and Extraction Process

Fruits of *S. cumini* were collected in December 2021 from the Medicinal Garden of Paranaense University (UNIPAR) (23°46.225′ S; 53°16.730′ W, 391 m), Paraná, Brazil. The botanical specimen was authenticated and deposited in the herbarium of the Medicinal Garden of Paranaense University (voucher number 53; SisGen registration: A672209). The fruit peels were manually separated, dried in a forced air circulation oven at 45 °C for five days, and pulverized using a knife mill (Usiram (METVISA, Brusque, Brazil)). 

The extract was obtained by infusing the pulverized peels in pre-boiling water. The extraction process lasted 5 h until the mixture cooled to room temperature. The solid residue was separated by filtration, and the filtrate was treated with ethanol to precipitate proteins and polysaccharides, resulting in a precipitate (EPI) and an ethanolic supernatant (ESI). The ESI was concentrated using a rotary evaporator, lyophilized, and stored at −20 °C until further analysis [24]. The extraction yield was determined as the ratio of the mass of the obtained extract to the mass of the dried peels, multiplied by 100.

### 2.2. Characterization of Extract

#### 2.2.1. Chromatographic Analysis

Phytochemical characterization of the extract was performed using ultra-high-pressure liquid chromatography (UHPLC), equipped with a BEH C-18 column (150 mm × 2.1 mm × 1.7 μm). The flow rate was maintained at 560 μL/min, and the column temperature was set at 60 °C. Mass spectrometry (MS) analysis used a quadrupole time-of-flight system (BRUKER (Billerica, MA, USA), Q-TOFII^®^ Billerica–USA). Both positive and negative ionization modes were employed, adhering to previously described methodologies [8]. Chromatograms were processed using MetaboScape software ((https://www.bruker.com/en/products-and-solutions/mass-spectrometry/ms-software/metaboscape.html, accessed on 27 September2024, Bruker®, Billerica, MA, USA), with spectral comparisons made against databases including Bruker MetaboBASE^®^ Personal Library 3.0, Bruker HMDB Metabolite Library 2.0, and Bruker MetaboBASE^®^ Plant Library (Billerica, MA, USA).

#### 2.2.2. Quantification of Total Phenolic Compounds

The total phenolic content (TPC) was quantified following the methodology proposed by Singleton and Rossi [25]. The extract, at a concentration of 1000 µg mL^−1^, was analyzed using a UV/Vis spectrophotometer (KASUAKI, São Paulo, Brazil) at 765 nm. A standard calibration curve (ranging from 3.90 to 250 µg mL^−1^) was constructed using gallic acid, resulting in the following linear equation: y = 14.269x + 65.544 (R^2^ = 0.9905). All measurements were performed in triplicate, and the TPC was expressed as micrograms of gallic acid equivalents per gram of extract (µg_GAE_ g_ext_^−1^).

#### 2.2.3. Antioxidant Capacity

The antioxidant capacity of the extract was evaluated using three different assays—the 2,2-diphenyl-1-picrylhydrazyl (DPPH) radical scavenging assay, the 2,2’-azinobis-(3-ethylbenzothiazoline-6-sulfonic acid) (ABTS^+^) assay, and the ferric reducing antioxidant power (FRAP) assay. The extract concentration used in each assay was 1000 µg mL^−1^, following the methodology described by Tominc et al. [9]. 

For the DPPH assay, a standard curve (1000 to 50 μM) was constructed using Trolox, yielding the linear equation y = −0.3925x + 473.92 (R^2^= 0.9914), with the results expressed in μM Trolox equivalents. For the ABTS^•+^ assay, a standard curve (2000 to 100 μM) was also prepared using Trolox (y= −0.2972x + 693.17; R^2^= 0.9982), with results expressed as μmol of Trolox per gram of extract (µmol_Trolox_ g_ext_^−1^). 

For the FRAP assay, ferrous sulfate (100–2000 µM) was used to create a standard curve (y = 0.6232x − 30.333; R^2^= 0.9982), and results were expressed as µmol of Fe^2+^ per gram of extract (µmolFe ^2+^ g_ext_^−1^). Quercetin (purity ≥ 95.0%, Sigma-Aldrich^®^ (Burlington, MA, USA)), at a concentration of 1000 µg mL^−1^, served as a positive control in these assays. All analyses were performed in triplicate.

#### 2.2.4. Antibacterial Activity

The antibacterial activity of the extract was assessed using the broth microdilution method in 96-well plates, following Clinical and Laboratory Standards Institute (CLSI) guidelines [26]. The bacterial strains tested included *Staphylococcus aureus* (ATCC 12026), *Streptococcus pyogenes* (ATCC 19615), *Staphylococcus epidermidis* (NEWP 0128), *Pseudomonas aeruginosa* (ATCC 9027), and *Escherichia coli* (ATCC 25922). The bacteria were cultured in brain heart infusion (BHI) broth, and standard suspensions were prepared at a concentration of 1.5 × 10^8^ CFU mL^−1^ in sterile 0.9% saline solution. 

The extracts were solubilized in sterile water and tested at concentrations ranging from 125 to 1.25 mg mL^−1^. The plates were incubated at 36 °C for 24 h. After incubation, 2% 2,3,5-triphenyl-tetrazolium chloride (TTC) (Sigma-Aldrich^®^ (St. Louis, MO, USA)) was added. The minimum inhibitory concentration (MIC) was determined as the lowest extract concentration that visibly inhibited bacterial growth. To establish the minimum bactericidal concentration (MBC), samples from wells showing no visible growth were plated on BHI agar and incubated at 36 °C for 24 h. The MBC was defined as the lowest extract concentration capable of preventing the appearance of colony-forming units.

### 2.3. Preparation of Film-Forming Solution

Phase A was prepared by dissolving the preservative methylparaben in water (q.s. 100%), which was heated to 80 °C to ensure solubilization. After cooling to room temperature, 4.5% poly (vinyl alcohol) (PVA) was dispersed in the solution and stirred continuously for 2 h. Concurrently, 4.5% polyvinylpyrrolidone (PVP) was dissolved in 17.5% absolute ethanol to form Phase B, while the extract (6.25%) was dissolved in 12.5% ethanol to form Phase C. Both Phases B and C were stirred at room temperature for 2 h before being gradually added to Phase A under constant stirring. Finally, a 0.3% imidazolidinyl urea solution (50%) was added, and the resulting FFS was stirred for 16 h at room temperature, as previously optimized [8]. 

A control FFS, devoid of *S. cumini* extract, was simultaneously prepared. All FFS formulations underwent physical and chemical characterization, with each analysis conducted in triplicate. The FFS developed was not compared to a positive control due to the current absence of a commercially available product with similar characteristics specifically intended for postoperative treatment.

### 2.4. Characterization of the Film-Forming Solution

#### 2.4.1. Organoleptic Properties

The organoleptic properties of the FFS were evaluated on the hands of three healthy volunteers, following the protocol approved by the Ethics Committee on Research Involving Human Beings of Paranaense University (approval number 6,301,818). Before the experiment, participants were informed about the purpose, methodology, and potential consequences of the analysis, and written consent was obtained. 

The evaluation included the formation and adhesion of the film, drying time, and post-drying appearance. The appearance, coloration, gloss, and transparency of the dried film were scored on a scale from 1 to 7. The following parameters for adhesion were used: 1—adhesive; 2—non-adhesive. The following parameters for appearance were used: 1—shiny and transparent; 2—transparent, but without glare; 3—transparent, but flaky; 4—whitish film; 5—ruddy and shiny; 6—reddish but dull; and 7—reddish, but scaly. The FFS *S. cumini* extract and the control FFS were evaluated in triplicate [27].

#### 2.4.2. Mechanical Properties

##### Folding Endurance

The mechanical resistance of the film was assessed by repeatedly folding the film at the same location until it broke or until 300 folds were reached, which is considered satisfactory performance. This test was conducted on three samples of each film [28].

##### Tensile Strength and Elongation

Tensile strength and elongation were measured using a universal testing machine (Biopdi (São Carlos, Brazil)) equipped with MBio-BioPDI software and a 100 N load cell. Three strips of each film formulation (60 × 10 mm) were fixed in grips set 30 mm apart and were subjected to tensile force at a speed of 15 mm/min.

#### 2.4.3. Barrier Properties 

##### Water Vapor Transmission Rate

The water vapor permeability (WVTR) of the films was determined gravimetrically at 25 °C, according to ASTM E96/E96M-22 standards [29]. The films were applied to permeation cells containing 50 mL of distilled water. These cells were then placed in desiccators containing blue silica gel and the assembly was stored in a BOD chamber at 25 °C. The films were monitored for mass gain by weighing for 24 h. The test was performed in triplicate.

##### Water Solubility

For the water solubility (WS) test, the films were initially dried for 24 h at 105 °C. Following drying, the films were weighed (Wi) using an analytical balance (GEHAKA^®^, model AG-200 (São Paulo, Brazil), then placed in Erlenmeyer flasks containing distilled water. The flasks were agitated in a shaker at 80 rpm and were maintained at 25 °C for 24 h. After this period, the films were dried again in an oven at 105 °C for 24 h to determine the final mass (Wf). WS was calculated using Equation (1).

WS = ((Wi − Wf)/Wi)(1)

#### 2.4.4. Morphology

The morphological structure of the control FFS and *S. cumini* FFS was analyzed by scanning electron microscopy (SEM) using a VEGA3 TESCAN (TESCAN^®^ (Brno, Czech Republic)), operating at an accelerating voltage of 10 kV. The films were dried in a desiccator and were freeze-dried using liquid nitrogen (N2). They were then placed on an aluminum tray and a thin layer of gold–palladium was deposited on the surfaces using a SC7620 sputtering device (Quorum Technologies^®^ (Puslinch, ON, Canada)) for 180 s to avoid the surface charge effect.

#### 2.4.5. pH

The pH of the formulation was measured using a calibrated digital potentiometer (ION^®^, pHB 500 (Araucária, Brazil)). The probe was immersed in the FFS, and readings were taken at 25 °C [30].

#### 2.4.6. Recovery Content

To evaluate the recovery content, 1 mL of FFS was added to 9 mL of phosphate-buffered saline (PBS, pH 7.4). The mixture was agitated at 250 rpm in a shaker (TECNAL^®^, TE-422 (Piracicaba, Brazil)) for 4 h to ensure complete extraction [30]. The samples were then filtered, and the TPC in the solution was analyzed using ultraviolet spectrometry. 

The recovery of the bioactive compounds released from the FFS was calculated by dividing the experimental concentration of TPC released in solution by the theoretical concentration of phenolics and multiplying by 100. The control formulation was used as a blank. All analyses were performed in triplicate.

#### 2.4.7. Delivery Volume

Ten measurements were taken using an analytical balance, after which the mean and standard deviation were calculated. The volume of solution delivered with each activation was calculated using Equation (2):

A_L_ = (W_t_ − W_0_) × D_n_ (2)

where A_L_ represents the volume of solution delivered per activation; W_t_ is the weight of the formulation after each activation; W_0_ is the initial weight of the formulation before activation; and D_n_ is the density of the formulation [15,31].

#### 2.4.8. Viscosity

Viscosity was determined using a Brookfield Digital viscometer (QUIMIS^®^, model Q860M26 (Diadema, Brazil)), with an accuracy of ±2.0% and a measurement range of 1 to 6,000,000 mPa·s at 25 °C. Spindle number 2 was used, with speeds ranging from 1 to 5 rpm. After spindle immersion in the formulation, the FFS was allowed to rest for 5 min before measurements commenced [32].

#### 2.4.9. Thermogravimetric Analysis 

The thermal stability of the films and extract was assessed using thermogravimetric analysis (TGA), employing a PerkinElmer STA 6000 simultaneous thermal analyzer. The analysis was conducted over a temperature range of 30 to 650 °C, with a heating rate of 10 °C min^−1^ and a nitrogen gas flow rate of 20 mL min^−1^ (99.999%, White Martins). The sample mass placed in the porcelain crucible was 10 mg.

#### 2.4.10. Fourier Transform Infrared Spectroscopy

The chemical structure of the control FFS and *S. cumini* FFS, along with any potential structural changes in these materials, was analyzed using Fourier Transform Infrared Spectroscopy (FTIR) with an attenuated total reflectance (ATR) accessory. The analyses were performed at 25 °C, within the spectral range of 4000 to 650 cm^−1^, with a resolution of 4 cm^−1^ and 9 scans, using a PerkinElmer FTIR spectrophotometer [33].

### 2.5. Statistical Analysis

The results were evaluated for analysis of variance (ANOVA). Mean comparisons were performed using Student’s t-test (*p* ≤ 0.05), using SPSS for Windows version 22.0 (SPSS Inc., Chicago, IL, USA).

## 3. Results and Discussion

This study aimed to characterize the extract of *S. cumini* and develop an FFS loaded with *S. cumini* extract with potential application for post-surgical therapies.

### 3.1. Yield and Characterization of S. cumini Extract

The drying process of *S. cumini* yielded 12.19%, with an extractive yield of 34.08%. The extraction yield of plant extracts is influenced by factors such as solvent alcohol content and extraction methods. Higher yields may indicate that the solvent successfully extracted a larger quantity of compounds from the plant matrix. However, a higher yield does not necessarily correlate with increased pharmacological activity, as part of the extract may consist of structural components derived from primary metabolism, which are not directly linked to biological activities [34,35].

In this study, the predominant compounds in the *S. cumini* bark extract were free flavonoids, followed by fatty acids and glycosylated flavonoids (Table 1). These findings align with previous research [36,37].

Flavonoids are critical antioxidants in neutralizing ROS and possess anti-inflammatory, immunomodulatory, and antimicrobial properties, providing significant pharmacological potential. The dual lipophilic and hydrophilic nature of flavonoids facilitates their penetration through the stratum corneum and their subsequent delivery to the dermis, where they can exert therapeutic effects through their antioxidant and anti-inflammatory actions [38].

Fatty acids, due to their affinity for the lipid layer of the stratum corneum, are incorporated into lipid bilayers, enhancing their diffusion into the deeper layers of the epidermis and dermis. In the dermis, fatty acids integrate into cellular membranes, playing a vital role in maintaining cellular structural and functional integrity [39]. Therefore, the chemical composition of the *S. cumini* extract supports its potential efficacy in topical applications, particularly in wound healing and other dermatological conditions [40].

Given the natural origin of the plant, variations in environmental factors, such as climate, can influence the qualitative and quantitative composition of these bioactives. While it is not feasible to obtain an extract with identical composition every time, the major classes of active ingredients, particularly flavonoids and fatty acids, remain consistent [41,42]. Therefore, while minor variations in the exact proportions may occur due to natural fluctuations, the core bioactive profile of the extract is preserved through standardized extraction and characterization processes.

Oxidative stress is a key factor in the inflammatory response, and the presence of antioxidants may foster a conducive environment for wound healing [43]. The antioxidant capacity of the *S. cumini* extract was assessed using DPPH, ABTS^+^, and FRAP assays (Table 2).

The antioxidant activity of *S. cumini* is attributed to the phenolic compounds present in the extract, with concentrations being consistent with those of prior studies [44,45,46]. Plant extracts with antioxidant properties have substantial potential for incorporation into formulations aimed at promoting wound healing and tissue regeneration. The inclusion of bioactive compounds in these formulations is intended to enhance biological functions, particularly antioxidant and antimicrobial activities [8]. The extract was further characterized by evaluating its MIC and MBC (Table 3).

Human skin serves as both a physical and immunological barrier and hosts a resident microbiota, which includes various bacterial species. Common pathogens such as *E. coli*, *S. aureus*, *P. aeruginosa*, *S. epidermidis*, and *S. pyogenes* are frequently found in wounds, surgical sites, and severe infections [47], justifying their inclusion in antimicrobial evaluations.

Table 3 shows identical values for all microorganisms, which is justifiable since the concentration was the same in all cases. Additionally, the MIC was equal to the MBC, indicating that the concentration required to inhibit bacterial growth was also sufficient to kill the bacteria.

This consistency in results can be attributed to the uniform efficacy of the antimicrobial agent used, which exhibited the same activity against all tested bacteria. The absence of variation in the data reinforces the reliability of the employed method and the reproducibility of the obtained results.

The antimicrobial activity of *S. cumini* has been confirmed by numerous studies [48,49,50,51,52,53], attributed primarily to its high concentration of phenolic derivatives. These results, combined with the extract’s antioxidant capacity, support the development of an FFS to aid in the postoperative healing process. The concentration of the extract used in the FFS was determined based on this evidence.

### 3.2. Physicochemical Analyses of the Formulations

To evaluate the physicochemical characteristics of the FFS formulations, an organoleptic analysis was conducted, with results detailed in the table below (Table 4).

There were no statistically significant differences in drying time among the developed FFS formulations. Both films exhibited adhesive properties, a crucial factor that ensures prolonged contact between the formulation and skin tissue, thereby optimizing the efficacy of active compound delivery to the application area [54]. Moreover, good adhesion minimizes the risk of transferring active compounds to other individuals or clothing [55].

The incorporation of *S. cumini* extract altered the film’s hue, resulting in a pale brown coloration. Despite this color change, the transparency of the films remained unaffected.

Table 5 presents the mechanical properties of the films, including folding endurance, tensile stress, and elongation, as well as barrier properties such as the WS and WVTR of the FFS. Mechanical tests indicated no significant difference in folding endurance behavior between the control FFS and *S. cumini* FFS. However, significant differences in tensile stress and elongation were observed, with *S. cumini* FFS exhibiting a greater tensile strength.

The enhanced folding resistance is attributed to the use of PVA polymer, which provides robustness and flexibility [56]. This property ensures the films are suitable for transdermal drug delivery, maintaining satisfactory mechanical properties, flexibility, and bioadhesion after application [55].

Regarding barrier properties, no significant changes were observed in the analyzed films. Both exhibited 100% WS, a critical feature for wound healing, as it ensures a structured matrix that facilitates the release of bioactives upon contact with the wound. Additionally, the high solubility contributes to maintaining structural moisture, aiding in film removal without causing abrasion to the affected area [57].

**Figure 1:**
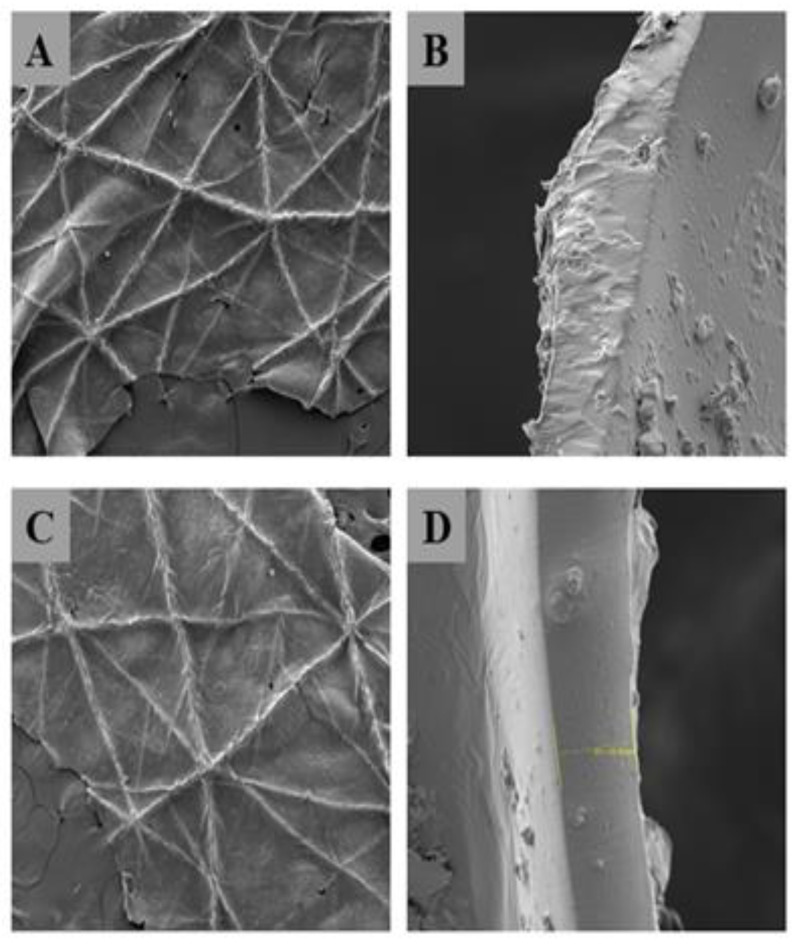
Scanning electron microscopy (SEM) images of (**A**) the surface morphology of control FFS; (**B**) the cross-sectional structure of control FFS; (**C**) the surface morphology of *S. cumini* FFS; and (**D**) the cross-sectional structure of *S. cumini* FFS.

To reinforce these observations, SEM analysis (Figure 1) provided a detailed examination of the surface and cross-sectional structure of the films. The observations revealed a homogeneous, compact structure devoid of pores, a characteristic attributed to the use of PVA, which controls water absorption and induces fiber swelling. This phenomenon results in reduced pore size and the observed low WVTR [58,59].

Surface images confirmed that the film conformed well to the skin, achieving perfect adhesion. No structural modifications were observed between the micrographs of the control FFS and *S. cumini* FFS.

Physicochemical characterization included the evaluation of pH, recovery content, and the volume of solution delivered to each actuation (Table 6).

The extract increased the pH of *S. cumini* FFS compared to the control FFS, closely resembling human skin pH [8]. Maintaining a compatible pH during the application of medicinal extracts is essential for preserving skin integrity and ensuring therapeutic efficacy. The skin’s acidic pH regulates barrier function, microbiome maintenance, and overall tissue health. Therefore, formulations with a pH similar to that of the skin may enhance penetration, efficacy, and compatibility, resulting in therapeutic benefits and the promotion of skin health [60].

The study revealed that the phenolic compound recovery content of *S. cumini* FFS is satisfactory (88.67% recovery content, corresponds to 5.57 mg of extract per mL of FFS), indicating the formulation’s efficacy in delivering bioactives, ensuring that the antioxidant and anti-inflammatory benefits of the extract are delivered in postoperative and therapeutic applications. This result is encouraging for the development of topical therapies aimed at wound healing and skin protection [61].

**Figure 2:**
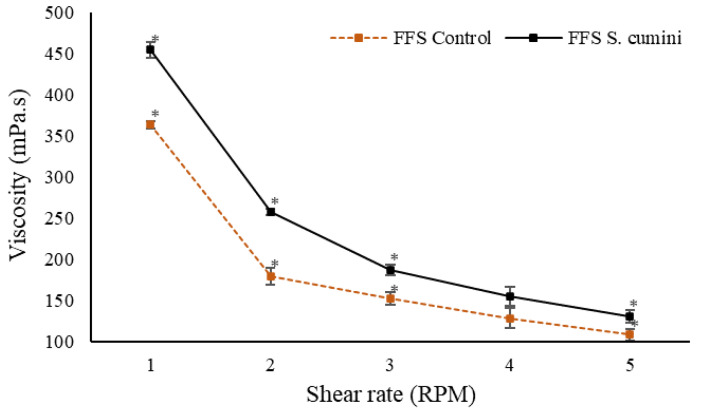
Flow behavior of control and *S. cumini* FFS. * *p* < 0.05 compared to the previous time with Tukey’s test.

The delivery volumes of FFS showed low variation and no significant differences between applied doses. The low variation is necessary for ensuring uniform therapeutic dosing and the concentration of delivered bioactives [8]. Additionally, rheological results (Figure 2) demonstrate the pseudoplastic flow behavior of the FFS, where an increased shear rate leads to the reorganization of the polymeric structure, resulting in subsequent shear occurring more rapidly and apparent viscosity decreasing [62]. This characteristic supports the delivery volume data and is important for the proper flow of FFS after valve actuation.

A high resting viscosity is essential for maintaining system stability, as formulations with a higher viscosity tend to be more structured [62]. A comparison of the formulations shows that *S. cumini* FFS exhibits a higher resting viscosity, likely due to the presence of phenolic compounds in the extract. Phenolic compounds are organic molecules that can increase fluid viscosity through intermolecular interactions with the formulation matrix [63], resulting in a greater resistance to flow.

**Figure 3:**
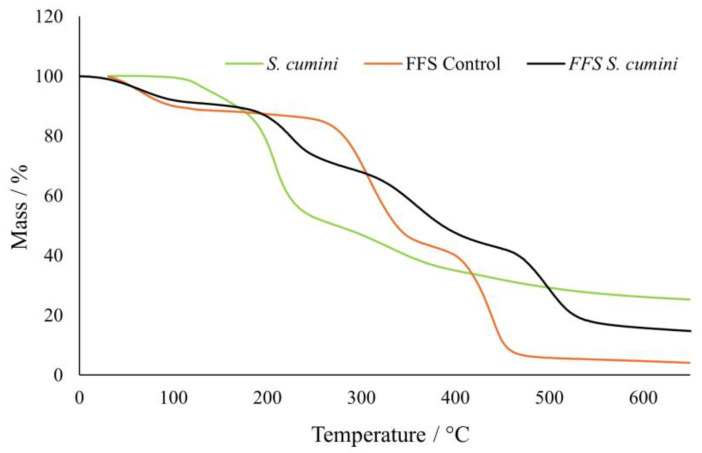
Thermogravimetric analysis (TGA) of FFS and *S. cumini* extract.

For the chemical characterization of the FFS, TGA was performed (Figure 3). The FFS formulations exhibit analogous curves due to their equivalent composition and preparation. However, a difference is notable from 210 °C, attributed to the presence of the extract.

Up to 280 °C, the control FFS shows slow mass loss, while the isolated extract exhibits significant loss starting at 140 °C. According to Paes et al. [64], this phenomenon suggests that the extract cannot withstand intense thermal processes. In the case of *S. cumini* FFS, significant mass loss begins at 210 °C, indicating that the thermal characteristics of the control FFS are altered due to interactions between the extract compounds and the polymer structure, mediated by hydrogen bonds [65], and demonstrating the extract’s protection during the thermal process when incorporated into FFS.

It is also notable that both formulations (control FFS and *S. cumini* FFS) exhibit their first thermogravimetric curve phenomenon around 50 °C, representing initial mass loss where water molecules are released [66]. The control FFS exhibits intense thermal alteration phenomena at 280 and 400 °C, likely indicating the structural decomposition of the polymer [66]. Similarly, Mallakpour and Mansourzadeh [67] reported that a PVA/PVP blend showed 50% mass degradation around 400 °C.

**Figure 4:**
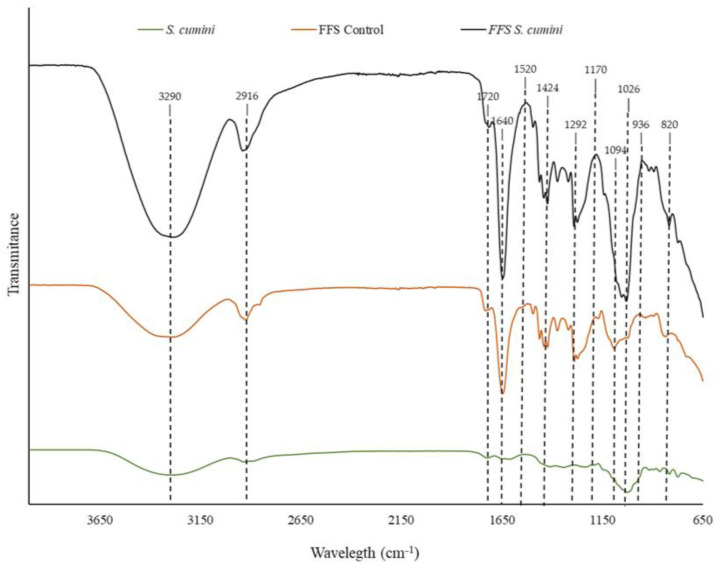
FTIR spectrum of FFS and *S. cumini* extract.

To assess potential interactions between the FFS components, FTIR analysis was conducted (Figure 4). 

Regarding *S. cumini*, the broad band at 3290 cm^−1^ corresponds to the hydroxyl group [68], which may be associated with the wide variety of polyphenols present in the extract. Additionally, the peak around 1026 cm^−1^ is also linked to bioactive compounds, indicating the presence of carbonyl (CO) stretching vibrations and single bonds between carbon and oxygen [69].

All samples exhibited a broad peak at 3290 cm^−1^, corresponding to hydroxyl hydrogen bonds; CH_2_ polymer stretching at 2916 cm^−1^; and the 1640 cm^−1^ peak associated with C=C bonding in PVA and PVP [66]. Other peaks of varying intensities were observed at 1720 cm^−1^, referring to the –CO stretching of PVA and PVP [70]; 1520 cm^−1^, associated with hydroxyl group stretching vibration in phenols [71]; 1026 cm^−1^, characteristic of C-OH stretching in PVA [72]; and 820 cm⁻¹, corresponding to CH_2_ stretching in PVA [73].

In the FFS samples, a peak around 1424 cm^−1^, related to the stretching of phenol and alcohol groups [74]; 1292 cm^−1^ to hydrocarbons in PVP [75]; 1170 cm^−1^ to carbon–oxygen bond stretching vibrations in the PVA structure [76]; 1094 cm^−1^ to PVA acetyl groups, specifically C–O stretching [66]; and 936 cm^−1^ to carbon stretching in PVP [77] were identified.

A comparison of the control FFS and *S. cumini* FFS demonstrated slight shifts in some peaks and changes in peak intensity, justified by intermolecular interactions. These changes may contribute to maintaining structure and increasing the films’ strength [8].

The *S. cumini* FFS represents a promising alternative approach for postoperative care. The results highlight the potential of *S. cumini* in accelerating wound healing and protecting against infections, offering a natural and effective solution. However, the scope was limited to the development and characterization of the FFS, encouraging future studies on stability, biodegradability, and in vivo efficacy evaluation.

## 4. Conclusions

The *S. cumini* FFS exhibited organoleptic and physicochemical characteristics compatible with topical application. Chemical and thermal analyses confirmed the formulations’ stability and the synergistic interaction between components, which is crucial for product efficacy. Incorporating *S. cumini* extract into the formulations significantly enhanced their properties, presenting relevant therapeutic potential. A promising effect on antioxidant protection was observed, contributing to ROS neutralization. Additionally, the extract demonstrated effective antimicrobial activity, standing out as a valuable component in the developed formulations.

## Figures and Tables

**Table 1:** Phytochemical characterization of *S. cumini* extract by UHPLC-MS.

Compounds	*m/z*	TR (Min)	µg g^−1^ *	% **
Isolinoleic acid	279.236	2.22	18,600	18.60
Chrysin	253.055	3.39	17,150	17.15
4-Malonyl ononin	267.069	3.59	8770	8.77
Sinapoyl malate-4′-methyl ester	353.069	4.22	7910	7.91
Scaposin	389.085	3.38	7560	7.56
Astragalin	447.088	16.09	7420	7.42
6-Hydroxyflavone	237.060	4.16	5870	5.87
Nevadensin	343.083	3.29	4870	4.87
2′-Methoxychrysin	283.063	3.34	4790	4.79
Epigallocatechin	305.066	17.34	4770	4.77
Medicarpin 3-O-glucoside-6′-malonate	269.083	3.58	3740	3.74
Pseudobaptigenin	281.048	3.93	2900	2.90
Neohesperidin	609.176	3.39	1930	1.93
Naringenin 7,4′-dimethyl ether	299.092	3.23	1030	1.03
Isosakuranin	447.132	3.36	780	0.78
Aspalathin	451.122	11.78	410	0.41
3,4-Dihydroxybenzaldehyde Protocatechualdehyde	137.023	3.58	390	0.39
Corymbosin	357.096	11.03	370	0.37
Robinetin	301.031	5.85	250	0.25
Isoxanthohumol	353.100	17.12	150	0.15
Kaempferide	299.057	3.21	150	0.15
Luteolin-4′-glucoside	447.092	15.32	120	0.12
Apiin	563.100	17.24	70	0.07
Flavonoids				54.79
Fatty acids				18.60
Glycosylated flavonoids				18.31
Cinnamic acid derivatives				7.91
Phenolic acids				0.39

* Concentration expressed as micrograms per gram of extract (µg g^−1^).** Compound percentages were calculated based on the total number of identified compounds.

**Table 2:** Quantification of total phenolic compounds, antioxidant capacity, and percentage comparison to the positive standard (quercetin).

Method.	Quercetin	*S. cumini*	%
TPC (µg_GAE_ g_ext_^−1^)	Nd	0.47 ± 0.17	-
DPPH (μM Trolox)	1085.82 ± 6.00 *	444.36 ± 14.71 *	40.92
FRAP (µmolFe ^2+^ g_ext_^−1^)	3378.85 ± 2.79 *	225.72 ± 4.04 *	6.68
ABTS^+^ (µmol_Trolox_ g_ext_^−1^)	3044.98 ± 0.01 *	175.53 ± 6.72 *	5.76

Results are expressed as mean ± standard deviation (*n* = 3). * Statistical significance (*p* ≤ 0.05) between control and *S. cumini* FFS (Student’s *t*-test). Quercetin was used as a positive control for antioxidant capacity, and the results for the extract were expressed by comparing the percentage (%) of activity as a function of the positive standard. nd: not determined.

**Table 3:** Minimum inhibitory concentration (MIC) and minimum bactericidal concentration (MBC) (mg mL^−1^) of *S. cumini* extract against different bacterial species.

Bacterial Strains	MIC	MBC
*E. coli*	62.5 ± 0.0	62.5 ± 0.0
*S. aureus*	62.5 ± 0.0	62.5 ± 0.0
*P. aeruginosa*	62.5 ± 0.0	62.5 ± 0.0
*S. epidermidis*	62.5 ± 0.0	62.5 ± 0.0
*S. pyogenes*	62.5 ± 0.0	62.5 ± 0.0

Results are expressed as mean ± standard deviation (*n* = 3).

**Table 4:** Organoleptic analysis of control and *S. cumini* FFS.

Test	Control FFS	*S. cumini* FFS
Drying Time ^a^ (min)	21 ± 3 *	29 ± 4 *
Adhesion	1	1
Appearance	1	5

^a^ Results are expressed as mean ± standard deviation (*n* = 3). * Statistical significance (*p* ≤ 0.05) between control and *S. cumini* FFS (Student’s t-test). As parameters for adhesion, (1) adhesive and (2) non-adhesive were used. Appearance: (1) transparent and shiny; (2) transparent, but without glare; (3) transparent, but scaly; (4) whitish film; (5) ruddy and bright; (6) ruddy, but dull; (7) ruddy, but scaly.

**Table 5:** Mechanical and barrier properties of control and *S. cumini* FFS.

Test	Control FFS	*S. cumini* FFS
Folding endurance (times)	>300	>300
Tensile stress (MPa)	3.06 ± 0.59 *	125.18 ± 4.28 *
Elongation at break (%)	12.25 ± 2.34 *	500.72 ± 17.11 *
WS (%)	100 ± 0	100 ± 0
WVTR (g/m^2^/dia)	6.03 ± 0.29	6.39 ± 0.04

Results are expressed as mean ± standard deviation (*n* = 3). * Statistical significance (*p* ≤ 0.05) between control and *S. cumini* FFS (Student’s t-test). Means of samples with absence of the sign (*) on the same line are not significantly different (*p* ≤ 0.05).

**Table 6:** pH, recovery content, and volume of solution delivered to each actuation of FFS.

Test	Control FFS	*S. cumini* FFS
pH	4.12 ± 0.03 *	5.53 ± 0.04 *
Recovery content (%)	0.00	88.67 ± 14.16 *
Delivered volume (mL)	0.16 ± 0.01	0.16 ± 0.00

Results are expressed as mean ± standard deviation (*n* = 3). * Statistical significance (*p* ≤ 0.05) between control and *S. cumini* FFS (Student’s t-test). Means of samples with absence of the sign (*) on the same line are not significantly different (*p* ≤ 0.05).

## Data Availability

Data are contained within the article.
